# Parenting styles and academic burnout in medical students: a moderated mediation model of resilience and stress

**DOI:** 10.3389/fmed.2025.1703534

**Published:** 2025-11-10

**Authors:** Chihao Teng, Liyang Shi, Chenchen Xu, Jianjun Zhu

**Affiliations:** 1 Second School of Clinical Medicine, Nanjing Medical University, Nanjing, Jiangsu, China; 2 Fourth School of Clinical Medicine, Nanjing Medical University, Nanjing, Jiangsu, China

**Keywords:** academic burnout, parenting styles, resilience, stress, medical students, conservation of resources theory

## Abstract

**Introduction:**

Amidst the confluence of post-COVID educational shifts, China’s New Healthcare Reform, and family structural changes driven by the two-child policy, academic burnout (ABO) severely impacts medical students’ health and performance. While parenting styles and resilience are recognized predictors, the dynamic interactions among parenting styles, resilience, and stress remain underexplored. This study, grounded in Conservation of Resources (COR) theory, investigates how parenting styles influence ABO through resilience, with stress as a moderator.

**Methods:**

A cross-sectional survey of 1,403 medical students from Eastern China was conducted in August 2025. Participants completed scales assessing parenting styles (s-EMBU), resilience (CD-RISC), stress (CPSS), and ABO (ABS). Data were analyzed using correlation, mediation (PROCESS Model 4), and moderated mediation analyses (PROCESS Model 14).

**Results:**

Contextual predictors of higher ABO included being a senior student, residing in a rural area, majoring in preventive medicine, and sleeping ≤7 h (all *p* < 0.05). Direct effects revealed that rejection (*β* = 6.331, *p* < 0.001) and overprotection parenting styles exacerbated ABO, whereas emotional warmth reduced ABO (*β* = −5.706, *p* < 0.001). Mediation analysis indicated that resilience mediated 44.2% (rejection), 74.8% (emotional warmth), and 41.4% (overprotection) of the total effects of parenting styles on ABO, with all 95% confidence intervals (CIs) excluding zero. Additionally, moderation analysis demonstrated that stress significantly undermined the protective function of resilience: at high stress levels (+1 SD), resilience’s protective influence on ABO was notably weaker (*β* = −0.239, 95% CI [−0.281, −0.198]), whereas under low stress conditions (−1 SD), resilience exerted a more robust reducing effect on ABO (*β* = −0.301, 95% CI [−0.343, −0.258]).

**Conclusion:**

Parenting styles influence ABO through resilience, a pathway dynamically moderated by stress. Precision interventions are proposed: resilience training for students with negative parenting histories and family resource repair for those from positive backgrounds under high stress. This framework synergizes resource optimization with resilience reinforcement to combat ABO.

## 1 Introduction

Since the COVID-19 pandemic began, online learning has evolved into a pivotal offshoot of traditional campus education, largely spurred by abrupt lockdown protocols. Concurrently, the crisis has left an indelible mark on medical students’ learning journeys—with the overwhelming majority navigating their studies predominantly through digital platforms (1). A defining advantage of online education lies in its temporal and spatial flexibility, a feature that not only upholds the continuity of instructional activities but also ensures students can progress steadily through theoretical curricula (2). That said, this shift in pedagogy has imposed significant strains on educational systems, with medical learners bearing disproportionate exposure to its drawbacks (3). Foremost among these challenges is diminished learning efficacy: studies highlight declines in academic achievement and waning student engagement with course material (4). Compounding this, a pandemic-era survey of medical students engaged in online learning found nearly half grappled with academic burnout (ABO) (5)—a phenomenon tightly intertwined with their psychological well-being (6). Although the pandemic has subsided, online teaching methods have become an integral component of medical education in the post-pandemic era, continuing to exert a notable influence on medical students. According to a recent systematic review, Chinese medical students’ burnout rate (55.1%) sits at the mid-to-high tier globally: lower than rates in Egypt (88%) and Saudi Arabia (80.7%) but significantly higher than Canada (34.2%). What distinguishes this cohort is that family cultural pressure (*β* = 0.42, *p* < 0.01) and amotivation (i.e., lack of intrinsic learning drive; *r* = −0.57) act as the core drivers of their burnout (7). This phenomenon not only affects students’ mental and physical health, leading to depressive, anxiety, and stress symptoms, but also impacts their academic performance, resulting in increased dropout rates and poor academic performance (7–10).

Multiple factors contribute to the academic burnout among medical students. According to a standardized study in Serbia, older age, frequent alcohol consumption, and sedative use are identified as key independent risk factors (11). In addition, interaction patterns are also important factors influencing medical students’ academic burnout, such as peer learning and teacher leadership (12, 13). Studies among Chinese medical students have shown that insufficient parental emotional support is significantly positively correlated with ABO (14). Senior medical students, due to the cumulative pressure of clinical training, exhibit significantly higher ABO levels than their junior counterparts (15). Medical students frequently grapple with substantial academic and emotional pressures, which often escalate into heightened stress levels and burnout. These challenges can exact lasting negative impacts that endure well beyond their training years, potentially contributing to suicidal ideation even as they enter professional practice (16). Consequently, the identification and implementation of effective strategies to mitigate these impacts are critical to fostering a healthier, more sustainable educational environment for medical students. Historically, scholarly inquiry into ABO related to medical students has largely centered on emotional and personality traits, whereas individual resources—examined through the lens of Conservation of Resources (COR) theory—have remained relatively underexplored (17–20). Building on this gap, the present study seeks to elucidate the mechanisms by which parenting styles and psychological resilience, conceptualized as composite individual resources, predict academic burnout from a COR theoretical perspective, combined with ecological systems theory and family systems theory. Additionally, it will examine the role of stress among these three variables based on the stress-vulnerability hypothesis.

It is essential to identify the factors that facilitate ABO. The implementation of China’s two-child policy has fundamentally reshaped family structures and parenting patterns, exerting dual effects on medical students who serve as the “firstborn” in their families. Tong et al. (21) revealed that firstborns in two-child families face compromised family functioning: Single-child families demonstrated significantly superior family atmosphere, personalization, and healthy family functioning rate. This disparity is partly driven by prevalent “parental preference” in two-child families, which may deprive firstborns of emotional support and amplify psychological distress. Contrastingly, Lao and Lin (22) documented an academic performance paradox: Children from two-child families outperformed only-children in standardized tests, attributed to sibling-enhanced willpower and extraversion. For medical students enduring rigorous training, policy-induced familial shifts—including diluted parental attention and reconfigured emotional support—may indirectly fuel academic burnout by eroding psychological resilience and sense of belonging. Critical gaps remain regarding how parenting styles mediate these dynamics under the policy, warranting targeted interventions to mitigate burnout risks. Bronfenbrenner’s ecological systems theory provides a pivotal framework for understanding the nested relationship composed of the factors. The theory conceptualizes individual development as “nested ecological systems,” where the microsystem, such as family and school, is the most proximal layer. What’s more, Family Systems Theory views the family as a dynamic, interconnected system of members, underscoring the reciprocal influences between individual behaviors and overall family functioning, with particular relevance to the impact of parental behaviors on child development. From the perspective of ecological systems theory, as the core of the microsystem, the family, through its parenting styles, shapes the early psychological resources (resilience) of medical students, thereby interacting with academic pressure and clinical practice demands in the school microsystem. Meanwhile, given the context of the COVID-19 pandemic under control—marked by reduced outdoor activity, diminished social engagement with friends, and heightened family interaction—positive parenting styles may exert a more pronounced influence on boosting medical students’ sense of career calling while alleviating their academic burnout. When parenting styles are unsupportive, students face greater difficulty coping with school-related stress, which further exacerbates ABO. This mechanism provides a theoretical foundation for the current study’s proposed “parenting style → resilience → ABO” mediated pathway. Existing research has suggested that college students’ academic burnout is influenced by multiple factors, including individual, interpersonal, school, family, and social aspects (23), among which parenting styles function as a core dimension of family factors (24). Parenting styles constitute a set of behaviors and patterns employed by parents during the process of raising children. Furthermore, this concept includes verbal and nonverbal interactions between parents and children in diverse situations. There are various parenting styles, such as positive parenting and unaffectionate parenting, which have been debated over the years. Among these, the Baumrind typology has achieved unparalleled prevalence worldwide (25). A recent study reveals that authoritative parenting is the most prevalent and significantly positively correlated with self-esteem among Pakistani medical students, with gender and age acting as moderating factors (26). Another study reveals that academic procrastination positively correlates with perceived stress and salivary *α*-amylase levels in Chinese medical freshmen, exacerbated by negative parenting styles, while positive parenting mitigates these effects (27).

According to COR theory, a recent study demonstrates that Chinese parents’ education anxiety triggers a resource loss spiral, leading to parental burnout. This state propagates to children’s academic burnout via emotional contagion and behavioral modeling, while high family functions as a critical resource buffer interrupts this anxiety-to-burnout pathway (28). In medical education, parenting styles, based on COR, act as the earliest environmental input, directly shape initial resource reserves (29). Supportive parenting (emotional warmth, autonomy encouragement) fosters psychological resources (high resilience, low anxiety) and social resources (family belongingness) through empathy and empowerment; in contrast, controlling or neglectful parenting (over-involvement, emotional detachment) restricts resource acquisition and may even deplete initial resources (reduced self-esteem) (25). Despite these insights, current research investigating the relationship between parenting styles and ABO among medical students remains limited, highlighting the necessity for further exploration of this association. Drawing on existing theoretical frameworks and accumulated literature, this study proposes Hypothesis 1: Parenting styles can directly predict academic burnout among medical students.

Resilience, in light of COR, is considered as a psychological resource, which has gained much attention in medical education (29). Connor and Davidson defined resilience as a personal quality that enables individuals to thrive amid adversities (30). A three-wave longitudinal study revealed that resilience psychological resilience significantly mitigated academic burnout from T1 to T2 on the early stage (31). Research has identified that individuals with high resilience exhibit traits such as perseverance, self-reliance, and equanimity, while also maintaining clear life goals to which they persistently strive even when confronting challenges or difficulties (32). Thus, as a critical personal psychological resource, resilience plays an essential role in helping individuals effectively cope with adversity and persist in pursuing their goals under stressful circumstances (33). COR theory posits that individuals with ample resources are more capable of effectively utilizing their current resources to secure additional ones (34). Resilience can be cultivated and strengthened through social mechanisms—for example, social support, constructive interpersonal relationships, and close interactions. Research involving nursing students indicates that supportive parenting styles help individuals internalize external social resources, which in turn bolsters their resilience (35). This predictive association between parenting styles and resilience has similarly been confirmed in studies of other college student populations (36). However, although existing evidence has clarified the association between parenting styles and resilience, the interaction effect of these two constructs in influencing ABO among medical students remains unexplored when they are conceptualized as composite coping resources. Building on prior research, where resilience functions as a mediating role in the effects of various psychological factors (37, 38), we propose Hypothesis 2: Among medical students, resilience mediates the relationship between parenting styles and academic burnout.

Stress is a major predictor of burnout (39). Lazarus and Folkman (40) defined stress as a “transactional process occurring when an event is perceived as relevant to an individual’s well-being, has the potential for harm or loss, and requires psychological, physiological, and/or behavioral efforts to manage the event and its outcomes.” Evidence suggests that medical students experience greater levels of stress and mental health issues than their peers majoring in other college disciplines (41) age-matched populations (42). Specifically, in China, the impact of New Healthcare Reform on the psychological well-being and career development of medical students has emerged as a critical concern in medical education research. Findings reveal a significant gap in policy awareness among students, with only 1.25% demonstrating “full understanding” of reform measures and 47.51% possessing superficial knowledge (43). This cognitive deficit amplifies anxiety over policy-driven challenges, including the abolition of drug markups and contract-based employment systems, contributing to elevated stress. Concurrently, escalating doctor-patient conflicts (72.01% linked to ethical lapses), fierce competition for employment (50.78% expressing career anxiety), and limited grassroots career appeal further erode students’ motivation and professional identity (44). Notably, the misalignment between educational frameworks and reform demands—such as deficient ethics education practices (only 73.72% supporting mentorship programs) and inadequate general practice training—exacerbates goal disorientation and adaptive difficulties. These interconnected factors form a multifaceted driver of stress. Under the background of New Healthcare Reform, a study categorizes stressors among medical graduate students into nine types, with their respective scores and characteristics outlined as follows. Clinical stress emerges as the primary stressor, with scores predominantly clustered between 5 and 8. Research stress, sharing the core status with clinical stress, also shows score concentrations in the 5–8 range. Employment stress exhibits the most dispersed score distribution, reflecting significant individual variability—a phenomenon linked to intensified job competition against the backdrop of enrollment expansion at key universities in 2024 and the scarcity of hospital positions. Academic stress ranks second in score dispersion, likely due to the vast medical knowledge system, heavy memorization burdens, and overlapping professional responsibilities. Economic stress, characterized by median scores indicating universality, stems from prolonged academic programs delaying financial independence, limited time for part-time work, and primary reliance on scholarships. Interpersonal stress arises from strong social needs during youth, which are often encroached upon by clinical/research commitments. Physical stress directly correlates with irregular sleep patterns (only 8.20% of students report regular routines). Doctor-patient stress, though scoring the lowest, displays gender differences, exacerbated by media portrayal and patients’ economic anxieties. Family stress registers the overall lowest scores, as most families do not impose coercive pressures (45). Notably, while this research finds minimal direct familial pressure on medical students, the interaction between family factors and other stressors in relation to academic burnout merits further investigation. Empirical findings indicate that such stress can exert a substantial influence on the ABO among medical students (46), with the cumulative burden of the stress often correlated with compromised resilience (4). Consistent with the loss spiral hypothesis COR theory, individuals may exhaust their resource reserves while managing stress and negative emotions stemming from life events. This depletion of resources, in turn, impairs their capacity to acquire new resources as a result of their diminished stores. Empirical research has demonstrated that resilience can substantially offset risks; however, its effectiveness is significantly weakened, or even rendered ineffective, when confronting greater or more intense stress (47). Consequently, we posit that the predictive influence of resilience on ABO among medical students may weaken when individuals are exposed to higher levels of stress. Stress might moderate the association between resilience and ABO. Drawing on this logic, we propose Hypothesis 3: In the mediated path of “parenting style → resilience → ABO,” the second-stage path “resilience → ABO” is moderated by stress—specifically, high stress weakens the negative predictive effect of resilience on ABO, whereas under low stress conditions, the buffering effect of resilience on ABO becomes stronger.

**Figure 1:**
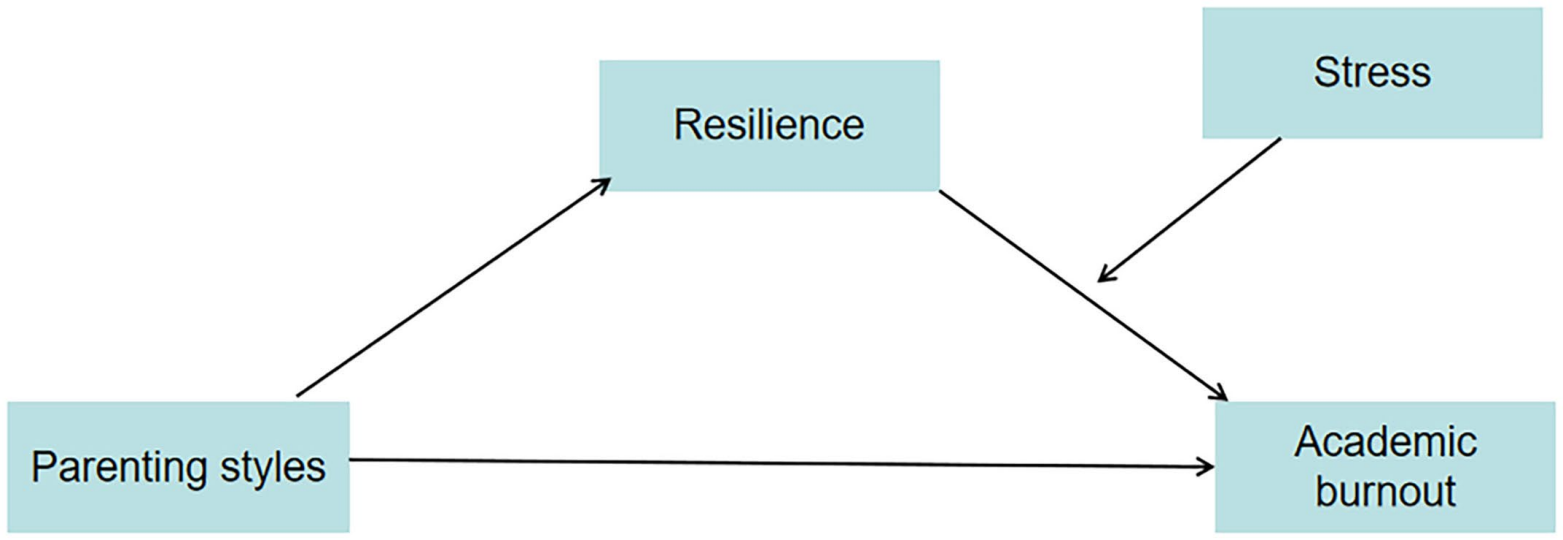
A moderated mediation model framework.

While extant literature has extensively scrutinized the nexus between family dynamics and academic burnout in medical students, the present cohort exhibits unique heterogeneity shaped by intersecting contextual factors. Under the dual backdrop of the post-COVID era and the New Healthcare Reform policy, coupled with the family structural shifts induced by China’s two-child policy, this study delves into the heterogeneous manifestations of medical student populations. The pandemic has catalyzed a paradigm shift toward online medical education, creating systemic challenges in learning adaptability and psychological resilience cultivation. The New Healthcare Reform has restructured the healthcare ecosystem, leading to divergent career expectation trajectories. Concurrently, the two-child policy has reconfigured intrafamilial resource allocation dynamics, engendering differentiated emotional support intensity and academic competition pressures between singleton and non-singleton children. These policy-induced intersections have resulted in multifaceted heterogeneities across stressor exposure (e.g., online learning ineffectiveness, occupational identity crises), resource endowments (e.g., familial emotional bonding quality, social capital accessibility), and coping strategy repertoires. Based on COR theory, ecological systems theory, family systems theory and the stress susceptibility hypothesis, along with extant research literature, this study developed a moderated-mediation model to examine the mediating role of resilience and the moderating role of stress in the relationship between parenting styles and academic burnout among medical students. The research framework is illustrated in Figure 1. The findings of this investigation aim to establish a theoretical framework to assist universities in designing targeted mental health curricula and intervention strategies that promote academic performance in medical students.

## 2 Materials and methods

### 2.1 Design

A descriptive, cross-sectional design was conducted to examine the relationship between medical students’ parenting styles, resilience, stress, and academic burnout.

### 2.2 Participants and procedures

A combined method of stratified random sampling and convenience sampling was employed. In August 2025, undergraduate students from four medical colleges/universities in eastern China were recruited for the survey. The institutions were divided into 3 strata based on their types: double first-class universities (36.1%, *n* = 506), regular universities (33.7%, *n* = 473), and vocational colleges (30.2%, *n* = 424), proportionate to their provincial medical student populations. Within each university, students were further stratified by grade, and 2 classes per grade were selected using the convenience sampling method with matched pairs (one standard class + one mixed-ability class; size difference ≤10 students) excluding specialized cohorts to participate in the survey. Inclusion criteria for study participants were: current students of medical colleges/universities and aged 18 or older. Ultimately, questionnaires were collected. We excluded 253 questionnaires that were not answered seriously and those with suspected extreme values. In total, 1,403 valid questionnaires were collected with an effective rate of 84.7%. A power analysis was conducted using G*Power 3.1 to determine the minimum sample size required for detecting a significant correlation. Assuming a small effect size (*r* = 0.10), a significance level of *α* = 0.001, and a desired statistical power of 0.90 (1−*β* = 0.90), the analysis indicated a minimum sample size of 287 participants. Our sample size reached the minimum sample size (48). All participating college students in this study signed informed consent forms, and the research was approved by the Ethics Committee of Nanjing Medical University [Approval No.145 NMULR (2025)].

### 2.3 Measures

#### 2.3.1 Parenting styles

The Simplified Parental Styles Questionnaire (s-EMBU) was developed by Arrindell et al. (79) through extracting 46 items from the standard version of the EMBU (Emotional Bonding in the Family Questionnaire) based on item content and psychometric indicators. This questionnaire comprises three subscales: rejection, emotional warmth, and overprotection. The Chinese version of the Simplified Parental Styles Questionnaire was revised by Jiang et al. (49), adopting a 4-point Likert scale ranging from 1 (never) to 4 (always). s-EMBU has been widely used in Chinese medical students and it has demonstrated good reliability and validity (50). Cronbach’s *α* coefficients for all dimensions of the original scale ranged from 0.70 to 0.84. In the present study, the Cronbach’s *α* coefficient of this scale ranged from 0.717 to 0.938.

#### 2.3.2 Academic burnout

The College Student Academic Burnout Scale (ABS), developed by Lian et al. (51) is one of the authoritative scales used to measure academic burnout among college students in China. The ABS comprises three dimensions: Inappropriate Behavior (6 items), Weak Sense of Achievement (6 items), and Low Mood (8 items). It employs a 5-point Likert scale, with scores ranging from 1 (Strongly Disagree) to 5 (Strongly Agree), and some items are reverse-scored. The total score of the scale is the sum of all item scores, ranging from 20 to 100. Higher scores indicate more severe academic burnout, with a score of ≥60 suggesting the presence of academic burnout. ABS has been widely used in Chinese medical students, and it has adequate reliability and validity (31). The Cronbach’s *α* coefficient of the scale was 0.93, and in the present study, its Cronbach’s α coefficient was 0.921.

#### 2.3.3 Resilience

The Connor–Davidson resilience scale (CD-RISC) was developed by Connor and Davidson in 2003, which includes 25 items and is divided into five dimensions: tenacity, tolerance of negative effects, positive acceptance of change, control and spiritual influences (30). The Chinese version of the CD-RISC was revised by Yu and Zhang (52), retaining 25 items of the original scale and adjusting it to three dimensions: tenacity, strength, and optimization. Tenacity (10 items) is retained from the original 5-factor model, strength (8 items) integrates the original Control and Positive Acceptance of Change dimensions, and Optimization (7 items) combines Spiritual Influences and Tolerance of Negative Effects. Five-point Likert scales were used, and each item was assigned according to the degree of conformity with the participants’ own situation (0 = never, 4 = almost always). CD-RISC has been widely used in Chinese medical students, and it has adequate reliability and validity (35). The internal consistency of CD-RISC (Chinese version) was tested in the general population in China (Cronbach’s *α* = 0.91) (52). The Cronbach’s α of the CD-RISC in the current study was 0.976.

#### 2.3.4 Stress

The Chinese Version of the Perceived Stress Scale (CPSS) was adapted from the original English Perceived Stress Scale (PSS) (44), a globally recognized and widely used instrument for assessing perceived stress. The PSS, initially developed in English, has been validated in multiple languages and comprises 14 items measuring feelings of stress and loss of control. Respondents rate their agreement with each item using a 5-point Likert scale (1 = Strongly Disagree to 5 = Strongly Agree), with some items reverse-coded. The CPSS was revised by Yang and Huang (80) and was tested in China (Cronbach’s *α* = 0.71) (45), retaining 14 items of the original scale. CPSS has been widely used in Chinese medical students, and it has adequate reliability and validity (20). In the present study, the Cronbach’s α coefficient of this scale was 0.898. The difference in Cronbach’s α between the original CPSS and this study is common, likely due to factors like a more homogeneous sample (medical students with shared stressors) and the focused nature of the measurement context, which aligns with psychometric expectations.

### 2.4 Data analysis

All the data were analyzed using IBM SPSS statistics 27.0 (IBM SPSS Statistics for Windows, IBM Corp., Armonk, NY, United States). The demographic characteristics of the participants were represented by descriptive statistics. Continuous variables were calculated from the mean and standard deviation, and intermittent variables were calculated over percentage and frequency. Normality testing was performed on continuous variables using the Kolmogorov–Smirnov test. We used Pearson correlation analysis to explore the relationship among parenting style, stress, resilience, and academic burnout. Harman’s single-factor test was used to evaluate the common method bias derived from self-reported data (53). The moderation and mediation effect was tested by PROCESS 4.1 (Model 14) developed by Hayes (54). It is assumed that the 95% CI does not contain zero, indicating that the effect is statistically significant. The report of this study is strictly in accordance with the STROBE Statement (55).

## 3 Results

### 3.1 Common method Bias test

Harman’s single-factor test was utilized to evaluate the influence of common method bias on the study findings (53). The results indicated that the first factor accounted for 32.69% of the variance, falling below the 40% threshold. This suggests that the common-method bias did not significantly impact the research results.

### 3.2 Descriptive statistics

The study included 1,403 medical students, with a balanced gender distribution (46.8% male, 53.2% female) as demonstrated in Table 1. Participants were predominantly undergraduates (29.8% freshmen, 22.5% sophomores, 19.3% juniors, and 38.4% seniors/specialty students). Clinical medicine (44.6%) and preventive medicine (17.5%) were the most common majors. An uneven urban–rural distribution was observed: 36.0% (*n* = 505) of the participants were from rural areas, while 64.0% (*n* = 898) were from urban areas and over half (52.1%) were only children. Parental educational backgrounds were concentrated at the high school to undergraduate levels (62% combined), while 57.1% of students reported no family members working in medicine.

**Table 1:** Sociodemographic characteristics and comparisons of total difficulties (*n* = 1,403).

Institution	*N*	%	Academic burnout			
			Mean	SD	*t*/*F*	*p*
Gender					0.754	0.451
Male	656	46.8	55.19	12.681		
Female	747	53.2	54.69	11.917		
Grade					2.541	0.038
Freshman	278	19.8	55.36	11.855		
Sophomore	316	22.5	55.16	13.031		
Junior	270	19.2	54.70	12.131		
Senior and above	539	38.4	56.33	12.136		
Institution					2.817	0.060
Double first-class initiative university	507	36.1	55.94	12.543		
Regular university	473	33.7	54.51	11.843		
Vocational college	423	30.1	54.18	12.383		
Major					6.623	<0.001
Basic medicine	53	3.8	57.60	12.412		
Clinical medicine	626	44.6	53.28	12.663		
Stomatology	103	7.3	53.86	12.360		
Preventive medicine	245	17.5	59.22	11.145		
Pharmacy	35	2.5	55.63	11.149		
Forensic science	47	3.3	53.53	11.677		
Medical technology	53	3.8	58.55	13.982		
Nursing	98	7.0	54.70	11.498		
Others	143	10.2	53.64	10.623		
Only child					−0.102	0.919
Yes	731	52.1	54.89	12.736		
No	672	47.9	54.96	11.770		
Class officer					−5.816	<0.001
Yes	883	62.9	53.50	12.372		
No	520	37.1	57.35	11.737		
Area					2.807	0.005
Rural	505	36.0	56.15	11.324		
Urban	898	64.0	54.24	12.738		
Parental education					0.443	0.875
Primary school	48	3.4	56.38	12.721		
Junior high school	238	17.0	55.42	11.731		
Senior high school	237	16.9	54.25	11.632		
Technical school	133	9.5	55.45	11.846		
Associate	233	16.6	54.66	11.770		
Undergraduate	404	28.8	54.61	12.619		
Master or doctor	92	6.6	55.52	14.802		
Not clear	18	1.3	57.00	15.278		
Parental occupation related to medicine					2.194	0.087
One parent related	137	9.8	55.55	11.393		
Both parents related	69	4.9	54.88	12.129		
Neither parent related with relatives related	396	28.2	53.60	12.818		
Neither parent related without relatives related	801	57.1	55.48	12.136		
Daily sleep hours (h)					2.917	0.004
≤7	875	62.4	55.67	12.273		
>7	528	37.6	53.70	12.200		
Annual household income (10,000 RMB Yuan)					2.194	0.087
<1	47	3.3	56.15	17.052		
1 ~ 5	175	12.5	56.10	11.448		
5 ~ 10	269	19.2	56.30	10.700		
10 ~ 15	279	19.9	55.11	11.977		
15 ~ 20	273	19.5	54.70	12.534		
20 ~ 25	158	11.3	53.21	11.920		
>25	202	14.4	53.18	13.688		

### 3.3 Univariate analysis

As shown in Table 1, it revealed significant differences in academic burnout across multiple factors. Specifically, senior students exhibited higher burnout levels (56.33 ± 12.14) compared to freshmen (55.36 ± 11.86; *F* = 2.541, *p* = 0.038). Preventive medicine (59.22 ± 11.15) and medical technology (58.55 ± 13.98) students reported significantly greater burnout than other majors (*F* = 6.623, *p* < 0.001). Class officers demonstrated lower burnout (53.50 ± 12.37) than non-officers (57.35 ± 11.74; *t* = −5.816, *p* < 0.001). Rural students (56.15 ± 11.32) showed higher burnout than urban counterparts (54.24 ± 12.74; *t* = 2.807, *p* = 0.005), and students sleeping ≤7 h daily (55.67 ± 12.27) reported more burnout than those with >7 h (53.70 ± 12.20; *t* = 2.917, *p* = 0.004). Among students whose parents were engaged in medical-related occupations, the mean ABO score was 55.48 ± 12.14; for those whose parents were not in medical occupations and had no relatives in the medical field, the mean ABO score was 53.60 ± 12.81 (*p* = 0.087). For students from households with an annual income >250,000 RMB, the mean ABO score was 53.18 ± 13.68, whereas for those from households with an annual income <10,000 RMB, the mean ABO score was 56.15 ± 17.05 (p = 0.087). These findings suggest that students from higher-income households and those from non-medical-background households may have lower ABO levels, which requires further validation in multivariate analysis.

### 3.4 Correlative analysis

**Figure 2:**
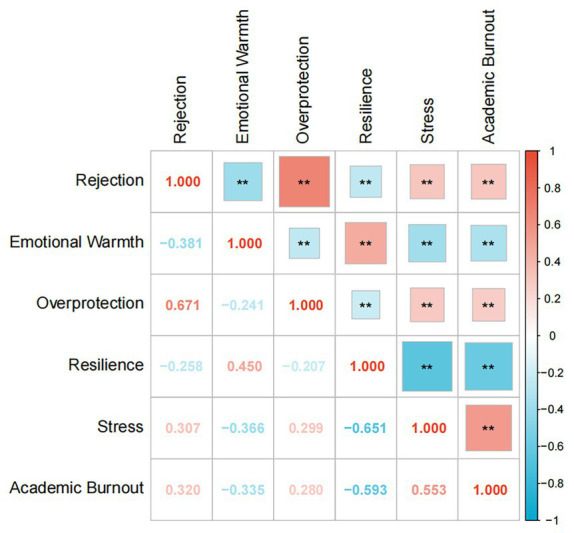
This heatmap illustrates Pearson correlation coefficients among six variables: rejection, emotional warmth, overprotection, resilience, stress, and academic burnout (diagonal values = 1.00, representing self-correlation). Cell values denote correlation coefficients (range: −1 to 1), with red (warm tones) indicating positive correlation, blue (cool tones) indicating negative correlation, and shade intensity reflecting correlation strength. ^**^Significant at the 0.01 level.

To examine the correlational relationships among the key study variables (parenting styles, resilience, stress, and academic burnout), Pearson correlation analysis was conducted, and the results are presented in Table 2, Figure 2. Specifically, regarding parenting styles and academic burnout (ABO), significant positive correlations were observed between both the Rejection parenting style (*r* = 0.320, *p* < 0.01) and the Overprotection parenting style (*r* = 0.280, *p* < 0.01) with ABO; conversely, the Emotional Warmth parenting style showed a significant negative correlation with ABO (*r* = −0.335, *p* < 0.01). Notably, perceived stress demonstrated the strongest positive correlation with ABO among the examined variables (*r* = 0.553, *p* < 0.01). Additionally, psychological resilience exhibited a strong negative correlation with ABO (*r* = −0.593, *p* < 0.01). Within the inter-correlations of parenting styles, the Rejection and Overprotection were positively correlated (*r* = 0.671, *p* < 0.01), and both were negatively correlated with Emotional Warmth (Rejection vs. Emotional Warmth: *r* = −0.381, *p* < 0.01; Overprotection vs. Emotional Warmth: *r* = −0.241, *p* < 0.01). Furthermore, resilience was negatively correlated with Rejection (*r* = −0.258, *p* < 0.01) and Overprotection (*r* = −0.207, *p* < 0.01) parenting styles, positively correlated with Emotional Warmth (*r* = 0.450, *p* < 0.01), and strongly negatively correlated with stress (*r* = −0.651, *p* < 0.01). Finally, stress showed positive correlations with Rejection (*r* = 0.307, *p* < 0.01) and Overprotection (*r* = 0.299, *p* < 0.01) parenting styles, and a negative correlation with Emotional Warmth (*r* = −0.366, *p* < 0.01).

**Table 2:** Correlations between parenting styles, resilience, and ABO.

Variables	1	2	3	4	5	6
1. Rejection	1					
2. Emotional warmth	−0.381^**^	1				
3. Overprotection	0.671^**^	−0.241^**^	1			
4. Resilience	−0.258^**^	0.450^**^	−0.207^**^	1		
5. Stress	0.307^**^	−0.366^**^	0.299^**^	−0.651^**^	1	
6. Academic burnout	0.320^**^	−0.335^**^	0.280^**^	−0.593^**^	0.553^**^	1
Mean	1.60	2.90	1.98	63.21	39.65	54.93
SD	0.62	0.71	0.67	17.90	9.10	12.28

The ABS scale has a total score range of 20–100, where higher scores indicate more severe ABO. In this study, the mean ABO score was 54.93 ± 12.28. ^**^Significant at the 0.01 level.

### 3.5 Mediation analysis

After finding internal links among parenting styles, resilience, and ABO, this study examined the potential mediating role of resilience between different parenting styles and ABO. In the Process macro proposed by Hayes and Scharkow (56), the mediating effect of learning anxiety was tested using Model 4. To achieve detailed insight into different parenting styles, the effect of resilience was explored between the three subscales of parenting style and ABO by bootstrapping.

Results from regression analyses in Table 3 indicated that rejection parenting style (*β* = 6.331, *p* < 0.001) significantly predicted higher academic burnout (ABO), while emotional warmth (*β* = −5.706, *p* < 0.001) showed a negative association with ABO. Overprotection (*β* = 5.120, *p* < 0.001) also positively predicted ABO. Notably, resilience mediated these relationships: rejection parenting negatively influenced resilience (*β* = −7.454, *p* < 0.001), and emotional warmth enhanced resilience (*β* = 11.198, *p* < 0.001), while overprotection reduced resilience (*β* = −5.528, *p* < 0.001). After accounting for resilience, direct effects of all parenting styles on ABO remained significant (rejection: *β* = 3.533, *p* < 0.001; emotional warmth: *β* = −1.440, *p* < 0.001; overprotection: *β* = 2.998, *p* < 0.001), suggesting partial mediation.

**Table 3:** Regression analysis of parenting styles on resilience and ABO in Chinese medical students.

Regression equation		Model Fit			Significance	
Outcome variables	Predictor variables	*R* ^2^	*F*		*B*	*T*
ABO	Rejection	0.102	159.582***	c_1_	6.331	12.633***
Resilience	Rejection	0.067	100.109***	a_1_	−7.454	−10.005***
ABO	Rejection	0.382	432.425***	c’_1_	3.533	8.204***
	Resilience			b_1_	−0.375	−25.164***
ABO	Emotional warmth	0.112	176.638***	c_2_	−5.706	−13.291***
Resilience	Emotional warmth	0.203	356.573***	a_2_	11.198	18.883***
ABO	Emotional warmth	0.358	390.052***	c’_2_	−1.440	−3.521***
	Resilience			b_2_	−0.381	−23.152***
ABO	Overprotection	0.078	118.845***	c_3_	5.120	10.902***
Resilience	Overprotection	0.043	62.799***	a_3_	−5.528	−7.925***
ABO	Overprotection	0.378	425.050***	c’_3_	2.998	7.599***
	Resilience			b_3_	−0.384	−29.564***

^***^Significant at the 0.001 level.

The total, direct and indirect effects are presented in Table 4. Bootstrap analyses confirmed the mediating role of resilience. For rejection parenting, 44.2% of its total effect on ABO was mediated by resilience (indirect effect = 2.798 vs. total effect = 6.331). Emotional warmth exhibited the strongest mediation (74.8%, indirect effect = −4.266 vs. total effect = −5.706), whereas overprotection showed a moderate indirect effect (41.4%, indirect effect = 2.122 vs. total effect = 5.12). These findings highlight distinct pathways: rejection and overprotection operate partly via resilience erosion or paradoxical enhancement, while emotional warmth primarily exerts protective effects through resilience strengthening. The differential effect sizes underscore the nuanced mechanisms by which parenting styles influence academic burnout.

**Table 4:** Total, indirect, direct effects, and effect size.

Types of parenting styles	Effect	Estimate	Boot SE	Boot CILL	Boot CIUL	Effective size
Rejection	Total effect	6.331	0.501	5.348	7.314	44.20%
	Indirect effect	2.798	0.445	2.000	3.725	
	Direct effect	3.533	0.431	2.688	4.377	
Emotional warmth	Total effect	−5.706	0.429	−6.548	−4.864	74.76%
	Indirect effect	−4.266	0.381	−5.041	−3.548	
	Direct effect	−1.440	0.409	−2.243	−0.638	
Overprotection	Total effect	5.120	0.470	4.199	6.041	41.45%
	Indirect effect	2.122	0.353	1.457	2.836	
	Direct effect	2.998	0.395	2.224	3.772	

SE, standard error; CI, confidence intervals; LL, lower limit; UL, upper limit.

### 3.6 Parenting styles and academic burnout: testing for moderated-mediation

This study employed hierarchical regression analysis to examine the moderated mediation effects of parenting styles on academic burnout, with all models demonstrating significant explanatory power (Δ*R*^2^ range: 0.043–0.203, Fs > 62.799, ps < 0.001). Specifically, all three parenting styles influenced academic burnout through the mediating role of psychological resilience, and the moderating effect of stress was significant across all models, though its direction and mechanism varied by parenting style. For rejecting parenting, parental rejection significantly and negatively predicted adolescents’ psychological resilience (*β* = −7.454, *t* = −10.005, *p* < 0.001)—indicating higher rejection corresponded to lower resilience—while psychological resilience itself significantly and negatively predicted academic burnout (*β* = −0.270, *t* = −14.644, *p* < 0.001), meaning stronger resilience was associated with lower burnout. Notably, the interaction between stress and psychological resilience (Stress × Resilience) significantly and positively predicted academic burnout (*β* = 0.003, *t* = 2.859, *p* < 0.01), suggesting stress weakened the protective effect of resilience: as stress increased, resilience’s ability to mitigate burnout diminished. In the case of emotional warmth parenting, emotional warmth significantly and positively predicted psychological resilience (*β* = 11.198, *t* = 18.883, *p* < 0.001), through its direct effect on academic burnout was non-significant (*β* = −0.266, *t* = −2.399, *p* < 0.05). Resilience remained a significant negative predictor of burnout (*β* = −0.266, *t* = −13.608, *p* < 0.001), but the stress-resilience interaction (*β* = 0.003, *t* = 2.898, *p* < 0.01) amplified this relationship: under high stress, emotional warmth enhanced resilience’s capacity to reduce burnout more prominently. Finally, overprotective parenting significantly and negatively predicted psychological resilience (*β* = −5.528, *t* = −7.925, *p* < 0.001)—higher overprotection corresponded to weaker resilience—and resilience still negatively predicted burnout (*β* = −0.278, *t* = −15.057, *p* < 0.001). However, the stress-resilience interaction (*β* = 0.003, *t* = 2.807, *p* < 0.01) further exacerbated this negative pathway: under high stress, overprotection weakened resilience to a greater extent, thereby increasing burnout more pronouncedly. The Results are shown in Table 5.

**Table 5:** The moderated-mediating effect of parenting styles on academic burnout.

Regression equation		Model fit		Significance	
Outcome variables	Predictor variables	*R* ^2^	*F*	*B*	*T*
Resilience	Rejection	0.067	100.109***	−7.454	−10.005***
ABO	Resilience	0.421	254.504***	−0.270	−14.644***
	Rejection			2.746	6.463***
	Stress			0.335	9.065***
	Stress × Resilience			0.003	2.859**
Resilience	Emotional warmth	0.203	356.573***	11.198	18.883***
ABO	Resilience	0.407	239.397***	−0.266	−13.608***
	Emotional warmth			−0.951	−2.399*
	Stress			0.371	10.019***
	Stress × Resilience			0.003	2.898**
Resilience	Overprotection	0.043	62.799***	−5.528	−7.925***
ABO	Resilience	0.417	249.603***	−0.278	−15.057***
	Overprotection			2.152	5.485***
	Stress			0.335	8.971***
	Stress × Resilience			0.003	2.807**

*Significant at the 0.05 level; **Significant at the 0.01 level; ***Significant at the 0.001 level.

**Figure 3:**
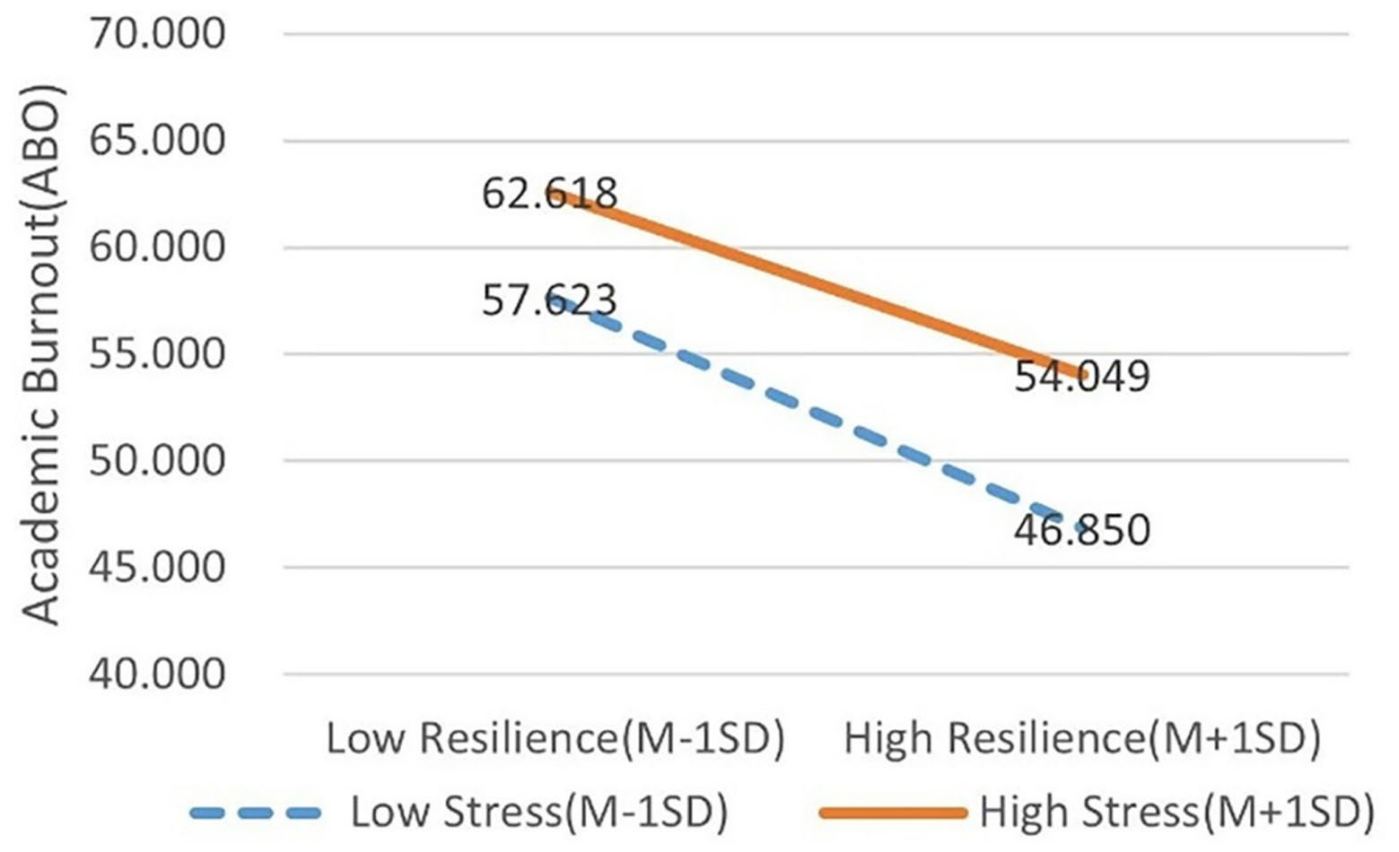
The unstandardized impact of resilience on academic burnout in different levels of stress. High stress level *β* = −0.239, low stress level *β* = −0.301.

After standardizing the study variables, the study divided the subjects into low (Z≦−1 SD) and high (Z≧1 SD) subgroups according to the standardized scores controlling for resilience for simple slope analysis. The study analyzed the moderating effect of stress on the relationship between resilience and academic burnout in the second half of the mediated model path. The simple slope test revealed that at the low stress level (−1 SD), the negative effect of resilience on ABO was *β* = −0.301 (95% CI [−0.343, −0.258]); at the high stress level (+1 SD), this negative effect weakened to *β* = −0.239 (95% CI [−0.281, −0.198]). The effect difference between the two levels was 0.062, indicating that the buffering effect of resilience decreased by approximately 20.6% when stress increased by 1 standard deviation. Figure 3 shows that the lower the level of stress, the greater the role resilience plays in relieving ABO.

The final step was to estimate the conditional indirect effect of parenting styles on ABO through resilience at three levels of stress (Table 6). For the rejection dimension of parenting styles, the indirect effect of rejection on academic burnout via resilience was significantly moderated by stress levels (−1 SD, Mean, +1 SD). Specifically, the indirect effect was 2.243 ([BootSE] = 0.407, 95% CI [1.496, 3.082]) at −1 SD stress; it decreased to 2.014 (BootSE = 0.363, 95% CI [1.35, 2.762]) at the mean stress level; and further reduced to 1.784 (BootSE = 0.361, 95% CI [1.123, 2.544]) at +1 SD stress. All 95% CIs excluded 0, indicating statistical significance across all stress levels, with the indirect effect progressively decreasing as stress levels rose. For the emotional warmth dimension, its indirect effect on academic burnout via resilience (characterized by a protective, negative direction) was also moderated by stress. At −1 SD stress, the indirect effect was −3.330 (BootSE = 0.407, 95% CI [−4.138, −2.54]); at the mean stress level, it was −2.976 (BootSE = 0.354, 95% CI [−3.685, −2.295]); and at +1 SD stress, it decreased to −2.622 (BootSE = 0.384, 95% CI [−3.379, −1.873]). These results suggest that the protective effect of emotional warmth—operationalized through its negative indirect effect on burnout—weakened as stress levels increased, with all 95% CIs excluding 0. For the overprotection dimension, its indirect effect on academic burnout via resilience (characterized by a detrimental, positive direction) was similarly moderated by stress. At −1 SD stress, the indirect effect was 1.705 (BootSE = 0.325, 95% CI [1.100, 2.36]); at the mean stress level, it was 1.537 (BootSE = 0.291, 95% CI [0.988, 2.131]); and at +1 SD stress, it decreased to 1.369 (BootSE = 0.284, 95% CI [0.847, 1.962]). This pattern indicates that the detrimental effect of overprotection—reflected in its positive indirect effect on burnout—diminished as stress levels rose, with all 95% CIs excluding 0. Collectively, these findings demonstrate that stress significantly moderates the association between family parenting styles and academic burnout through its influence on the mediating role of resilience. Specifically, all three parenting style dimensions (rejection, emotional warmth, overprotection) exhibited stress-dependent indirect effects on academic burnout via resilience, with each effect reaching statistical significance (as indicated by non-zero 95% CIs).

**Table 6:** The moderation effect of stress.

Dimension	Stress level	Effect	Boot SE	Boot LLCI	Boot ULCI
Rejection	−1 SD	2.243	0.407	1.496	3.082
	Mean	2.014	0.363	1.350	2.762
	+1 SD	1.784	0.361	1.123	2.544
Emotional warmth	−1 SD	−3.330	0.407	−4.138	−2.540
	Mean	−2.976	0.354	−3.685	−2.295
	+1 SD	−2.622	0.384	−3.379	−1.873
Overprotection	−1 SD	1.705	0.325	1.100	2.360
	Mean	1.537	0.291	0.988	2.131
	+1 SD	1.369	0.284	0.847	1.962

## 4 Discussion

Against the intersecting backdrops of the post-COVID era, the New Healthcare Reform policy, and family structural transformations driven by China’s two-child policy, this study examines the heterogeneous manifestations within medical student populations. The pandemic has accelerated a paradigm shift toward online medical education, posing systemic challenges to students’ learning adaptability and the cultivation of psychological resilience. Meanwhile, the New Healthcare Reform has reshaped the healthcare ecosystem, resulting in divergent trajectories of career expectations among medical students. Concurrently, the two-child policy has reconfigured dynamics of intrafamilial resource allocation, giving rise to differentiated intensities of emotional support and varying academic competition pressures between singleton and non-singleton children. These policy-induced interactions have engendered multifaceted heterogeneities across three dimensions: exposure to stressors, access to resources, and repertoires of coping strategies. Guided by the COR theory, ecological systems theory, family systems theory, and the stress susceptibility hypothesis, this investigation developed a moderated mediation framework and applied it to a sample of medical students navigating the complexities of this era. Focusing on the association between parenting styles and academic burnout, the study examined the mediating role of resilience while exploring the moderating effect of stress. The findings not only broaden the applicability of these theoretical models but also deepen understanding of the critical mechanisms through which parenting styles influence academic burnout among medical students. In addition, grade, major, class officer experience, area and daily sleep hours all had a significant impact on the level of academic burnout of medical students. Overall, the results of this study validated the proposed hypothesis. The results offer actionable insights to inform targeted interventions for alleviating academic burnout in this population.

In this study, the total score of academic burnout was 54.93 ± 12.28, which was similar to the research results of Yang et al. (14). Senior students exhibit significantly higher levels of ABO compared to lower-grade students (15). This discrepancy is primarily attributed to the cumulative nature of stressors and the lagged development of psychological resilience. Senior students must simultaneously cope with triple stressors: theoretical exams, clinical internships and rotations, and future planning (postgraduate entrance exams/employment). Compared to the “basic course learning” stressor of lower grades, senior students face more complex and intense stressors. According to the COR theory, the “resource depletion” caused by stress accumulates over time, while the development of psychological resilience (e.g., problem-solving abilities, emotional regulation) requires gradual accumulation through experience. This results in a more pronounced imbalance between stress and resilience among senior students (57). Additionally, senior students begin clinical rotations, transitioning from “passive learners” to “active participants.” Role ambiguity (identity conflicts between “medical student” and “prospective doctor”) further intensifies their stress perception (58). Additionally, upper-year medical students—having weathered extended stretches of pandemic-induced lockdowns—exhibit heightened susceptibility to academic burnout, with the prolonged nature of these disruptions amplifying their stress and eroding engagement over time (59). Table 1 shows that students majoring in Preventive Medicine/Medical Technology exhibit significantly higher levels of ABO compared to those in Clinical/Dental and other majors. This may be linked to employment attitudes and expectations. A study indicates that 18.5% of preventive medicine students believe the pandemic has negatively impacted employment (e.g., unstable job demand, increased occupational risks), and these students report significantly higher stress levels (*β* = 2.56, *p* < 0.001). Additionally, 12.5% of students have considered changing careers due to pandemic-related concerns, primarily fearing restricted career prospects or overwhelming workloads (60). According to Person-Environment (Fit P-E Fit) theory and Self-Determination Theory (SDT) theory, it is inferred that this discrepancy is also linked to the heterogeneity in professional training objectives and perceived professional values. Notably, in China, Clinicians generally enjoy higher social status than Preventive Medicine/Medical Technology professionals. Preventive Medicine focuses on public health practice (e.g., epidemiological surveys, vaccination), while Medical Technology emphasizes skill-based operations (e.g., medical imaging, laboratory testing). In contrast, Clinical majors center on “disease diagnosis and treatment.” Daily tasks for Preventive Medicine/Medical Technology students (e.g., rural sampling, equipment maintenance) are often perceived as “low-technical-content” or “repetitive labor,” leading to lower perceived “competency-task fit” and consequently, exacerbated burnout (61). Conflict between family or social expectations and professional reality weakens their intrinsic motivation, ultimately leading to elevated ABO levels (62). Furthermore, during and beyond the COVID-19 pandemic, preventive medicine students have taken on increased frontline duties—including community epidemic control, epidemiological tracing, and vaccination campaigns—exposing them to heightened infection risks. Meanwhile, the pandemic has pushed these students to transition from basic medical research to studies focused on emergency management of public health incidents, yet accessing relevant scientific research resources has grown notably more arduous (63). This also accounts for the amplification of ABO.

This study found for the first time that parenting styles significantly predict academic burnout in medical students, which was consistent with previous findings in middle school students (64). The findings align with COR theory’s emphasis on resource dynamics. According to COR theory, individuals are motivated to allocate and utilize their resources efficiently. The availability of an initial resource surplus encourages individuals to maximize the utility of these resources and invest them in subsequent activities (65). Authoritative parenting, characterized by emotional warmth, was negatively correlated with burnout, corroborating studies showing that supportive family environments foster resilience and reduce stress vulnerability (26, 29). Conversely, rejecting and overprotective parenting styles exacerbated burnout, consistent with research linking controlling parenting to maladaptive coping strategies and emotional depletion (27). These results highlight the critical role of family resources in shaping students’ capacity to withstand academic pressures. From an ecological perspective, the family, as the earliest “microenvironment” encountered by medical students, shapes the mesosystem through interactions with other microenvironments, thereby influencing their academic outcomes (66, 67). What’s more, family systems theory underscores the interdependence between parental behaviors and student outcomes. Medical students face unique stressors, such as rigorous coursework and clinical responsibilities, which may amplify the impact of family on resource accumulation. Research on the relationship between parenting styles and academic burnout remains underexplored, whereas studies examining family functioning and academic burnout have been extensively conducted across diverse cultural groups. Notably divergent effect sizes emerged. Among Chinese medical students, family functioning demonstrated the strongest direct negative predictive effect on academic burnout (*β* = −0.963), underscoring the centrality of familial influence in collectivist cultures (68). In European graduate students, a moderate effect size (*β* = −0.28) highlighted the dispersed nature of social support networks in individualist societies (69). For Spanish adolescents, family functioning indirectly alleviated burnout by reducing emotional exhaustion (*β* = −0.23) and cynicism (*β* = −0.27), also indicating moderate effects (70). Consequently, culturally tailored interventions are pivotal for mitigating academic burnout. Collectivist cultures require restructuring family-centered emotional support systems. Individualist cultures must enhance the resilience of external support networks. Within China’s collectivist cultural context, investigating the impact of parenting styles on academic burnout holds significant practical relevance for developing targeted educational and psychological interventions. Meanwhile, although the relationship between parenting styles and academic burnout has received limited empirical attention within the medical student population, its associations with other comparable variables have been extensively explored in existing research. Parenting styles influence the development of medical students through three pathways: psychological, behavioral, and physiological. Authoritative parenting, characterized by high responsiveness and reasonable demandingness, significantly enhances medical students’ self-efficacy and resilience while reducing academic procrastination and depression risk (*β* = −0.32, *p* < 0.001) (26, 35). This parenting style buffers the negative impact of academic stress on mental health by promoting positive coping strategies and goal management abilities (35). Conversely, authoritarian parenting—marked by low care and high control—inhibits autonomous decision-making, directly increasing depression risk (odds ratio [OR] = 7.54) and procrastination behavior (correlation coefficient [*r*] = 0.23). It also triggers delayed stress responses by activating the sympathetic nervous system (evidenced by elevated salivary alpha-amylase [sAA] levels) (27, 71). Permissive and neglectful parenting, due to their lack of boundary setting, lead to deficits in time management skills and are particularly associated with low self-esteem (*r* = −0.29) among male medical students (26, 27). Cultural context also moderates these effects. In Eastern collectivist cultures, affectionate constraint from fathers correlates positively with suicidal ideation (OR = 3.09) (71), whereas some Asian groups (e.g., Pakistani men) exhibit greater adaptability to authoritarian parenting (26); in Western cultures, the positive impacts of authoritative parenting are more consistently observed (71). Educational interventions should incorporate cultural sensitivity: Families should adopt communication patterns characterized by “high responsiveness and moderate demandingness,” while institutions should screen high-risk students and provide cognitive-behavioral therapy (CBT) and resilience training (27, 35). In all, interventions targeting parenting styles could indirectly reduce burnout. For instance, parent-education programs emphasizing emotional warmth and autonomy support might empower students to develop adaptive coping mechanisms.

In addition, another finding of this study is that resilience is an intrinsic mediating mechanism that links parenting styles to the ABO of medical students. Both resilience and positive parenting styles are crucial psychological resources for individuals, and the significant positive relationship between these two constructs has been examined in various studies (72, 73). From the perspective of “Resource Caravans and Resource Pathways” in COR theory, dynamic management and flow of resources are a key determinant of individual adaptation outcomes (29). As a resource hub, psychological resilience, by integrating external inputs and internal regulation, becomes a critical mediating variable linking parenting styles and academic burnout. Moreover, research across various population has implied a negative effect resilience has on academic burnout (39, 74). Notably, a longitudinal study revealed that the temporal association between ABO and resilience was reciprocal and there exists a change in the relationship form bidirectional to unidirectional (31). These findings align with “resource loss spiral” and “resource gain spiral” in COR theory. This is manifested in the fact that academic frustration, as a form of long-term resource depletion, may lead to a decline in psychological resilience, resulting in a “resource spiral loss” (31); whereas high psychological resilience, through resource protection, blocks the developmental path of burnout, thereby fostering a “resource spiral growth” (75). This proposal suggests that implementing intervention strategies to enhance medical students’ resilience could serve as a valuable approach to counteract the detrimental effects of academic burnout. Empirical evidence from intervention studies underscores the potential of resilience—regarded as a critical psychological resource—to alleviate psychological distress among medical students and boost their subjective well-being (19, 20). Consequently, medical institutions may consider introducing resilience-building initiatives, such as mindfulness-based interventions and yoga training programs, to cultivate students’ resilience. Such efforts aim to support them in managing stress and reducing burnout.

Furthermore, the findings of this study reveal that among the multiple pathways via which parenting styles exert their mediating effects on ABO through resilience, the second path is subject to moderation by stress. Aligned with the stress-vulnerability hypothesis, our results demonstrate that resilience serves as a more robust negative predictor of ABO among individuals experiencing lower stress levels compared to those with higher stress. It indicates that, akin to other individual characteristics, resilience may not suffice to shield individuals from maladaptive outcomes in high-stress scenarios. This phenomenon may stem from the observation that people in lower-stress environments tend to display greater resilience and demonstrate superior proficiency in effectively harnessing their psychological resources. Within such contexts, resilience is more likely to relieve ABO. Consequently, stress act as risk factors that diminish the capacity of resilience to relieve ABO, thereby underscoring its role as a determinant of stress susceptibility and further corroborating the stress-vulnerability hypothesis (4, 47). Consistent with COR theory’s dynamic resource spirals, resilience mediates the protective effect of emotionally warm parenting on ABO more strongly under low stress, reflecting COR’s gain spiral stability, while its role in exacerbating ABO through rejection/overprotection parenting is more pronounced under low stress but attenuated under high stress, indicating an interruption of COR’s loss spiral. Our findings further indicate that, low stress enables effective resource utilization, enhancing resilience’s protective capacity; high stress, however, disrupts gain spirals in warmth-based pathways while buffering loss spirals in negative parenting contexts. This phenomenon—where high stress reduces the detrimental impact of negative parenting (rejection/overprotection) on academic burnout—can be explained through dual mechanisms derived from COR theory. First, according to resource allocation prioritization, high stress triggers survival-oriented resource reallocation (29), automatically diminishing attention to non-critical stimuli, thereby attenuating the perceived intensity of negative parenting. For instance, medical students during clinical exam periods may cognitively filter out parental criticism, reducing emotional resource depletion. Second, in light of partial interruption of loss spirals, stress activates compensatory resources (e.g., peer support, academic goal-driven motivation) to offset familial resource deficits, disrupting the “negative parenting → resource depletion → exacerbated ABO” spiral (65). This dynamic explains the observed decrease in indirect effects under high stress (Rejection: 2.24 → 1.78; Overprotection: 1.70 → 1.37), wherein stress-induced resource substitution mitigates the typical loss spiral. These dynamics underscore stress as a contextual determinant of resilience efficacy. Medical students frequently encounter distinct challenges, such as rigorous academic requirements, employment-related pressures, and uncertainties regarding career trajectories. Such challenges can induce stress, which exerts a substantial impact on their mental health and overall well-being (76). Notably, the prevalence of burnout among medical students has been documented, with studies indicating that nearly half of medical students experience moderate to high levels of burnout, which can be exacerbated by stress (77, 78). These findings highlight the significance of acknowledging the unique challenges encountered by medical students, as well as the potential for stress to impede their capacity to effectively utilize resilience. To address the distinct pathways through which parenting styles influence ABO, interventions should be tailored to each context: for negative parenting (rejection/overprotection), resilience training (e.g., mindfulness) can leverage high stress’s buffering effect to consolidate the interrupted loss spiral, wherein stress naturally mitigates resource depletion; meanwhile, for warmth-based parenting, targeted efforts to mitigate stress-induced resource depletion (e.g., family communication) aim to restore the attenuated gain spiral, countering the weakened protective effect of resilience under high stress. This approach aligns COR-based resource optimization with stress-contextualized resilience reinforcement, offering a precision framework for ABO mitigation.

### 4.1 Limitations

When interpreting these findings, several important caveats warrant attention. First, the cross-sectional design of the current study limits our capacity to determine directional relationships, temporal order, or causal associations between variables. Unlike longitudinal designs that track changes over time, cross-sectional data capture only a single snapshot of variables at a specific moment, making it impossible to determine whether parenting styles precede and predict academic burnout, or if burnout symptoms retroactively influence parental behaviors (e.g., parents may adjust their parenting in response to perceived student distress). This ambiguity risks conflating correlational patterns with causal directionality—a limitation that undermines the robustness of inferences about the “parenting style → resilience → burnout” pathway. Longitudinal or experimental designs in future research would be valuable to more thoroughly investigate potential causal mechanisms. Second, the sample was restricted to participants from Jiangsu Province, China; consequently, the extent to which these findings can be generalized to students in other provinces or cultural contexts remains unclear. Jiangsu’s advanced medical resources may boost family parenting’s buffering of academic burnout via clinical practice, identity, and support. Yet its generalizability is limited by China’s urban–rural medical resource gaps—underdeveloped regions lack training bases and faculty. Future studies must test this in resource-scarce areas, examining how urban–rural disparities moderate parenting’s role: rural students may see “family-school” support weaken amid resource pressures, while urban peers face new stress from parental over-investment. Clarifying regional heterogeneity is key to tailored burnout interventions and avoiding one-size-fits-all policies. Therefore, expanding recruitment to include a larger, more demographically diverse sample in future investigations would help enhance the generalizability and representativeness of the results. Third, the current study relied heavily on self-report instruments to assess academic burnout. Self-report measures, though convenient, have flaws. Biases (social desirability, recall) distort variable relationships, and they miss burnout’s multidimensionality—self-enhancement may cause gaps between reported burnout and actual impairment, weakening links to parenting styles/resilience. Subsequent studies should further explore the associations between parenting styles and diverse types of academic burnout to offer a more holistic understanding of these relationships. Future studies should also incorporate multi-source data (e.g., teacher-rated resilience, objective academic performance) and control for method factors via SEM to strengthen validity.

## 5 Conclusion

This study, grounded in COR theory, ecosystem theory, and family systems theory, demonstrates that parenting styles significantly influence ABO among medical students through psychological resilience, with stress acting as a key moderator. Emotionally warm parenting reduces ABO by enhancing resilience, while rejecting and overprotective styles exacerbate it by depleting resilience.

Crucially, stress differentially moderates these mediated pathways. For emotional warmth parenting, high stress mitigated resilience’s protective effect, with the indirect effect weakening from −3.33 (low stress) to −2.62 (high stress), indicating a disruption of COR’s gain spiral where stress depletes family-derived resources. Conversely, for rejection/overprotection parenting, high stress slows resource depletion, decreasing the indirect effect from 2.24 to 1.78 (rejection path) and from 1.70 to 1.37 (overprotection path). This reflects a partial interruption of COR’s loss spiral. These differential effects provide actionable strategies for medical educators. For emotional warmth pathways, efforts should focus on mitigating stress-induced resource depletion—such as through family communication workshops—to restore the “gain spiral” of resource accumulation, where stress would otherwise deplete family-derived supportive resources. Meanwhile, for rejection/overprotection pathways, leveraging stress-buffering effects via resilience training (e.g., mindfulness) can prevent relapse into the “loss spiral” of resource depletion, as high stress naturally slows but does not eliminate this harmful cycle. Together, these strategies form a theoretically coherent framework: by synchronizing stress-contextualized resource optimization (repairing depleted resources in warmth pathways and consolidating buffered resources in rejection/overprotection pathways) with targeted resilience reinforcement, we can systematically combat academic burnout at both the familial and individual levels.

## Data Availability

The datasets presented in this article are not readily available because the data involve sensitive personal and psychological information from medical students, and participants did not consent to public sharing of their individual responses. Besides, the study was approved by the Ethics Committee of Nanjing Medical University under approval number 145 NMULR (2025), which includes clauses protecting participant confidentiality. Requests to access the datasets should be directed to Nanjing Medical University.
